# The Buzz about Honey Bee Viruses

**DOI:** 10.1371/journal.ppat.1005757

**Published:** 2016-08-18

**Authors:** Laura M. Brutscher, Alexander J. McMenamin, Michelle L. Flenniken

**Affiliations:** 1 Department of Plant Sciences and Plant Pathology, Montana State University, Bozeman, Montana, United States of America; 2 Department of Microbiology and Immunology, Montana State University, Bozeman, Montana, United States of America; University of Kentucky, UNITED STATES

In this short review, we present our current understanding of the role of viruses on honey bee health and address some overarching questions in honey bee virology.

## Why Should I Be Concerned about Honey Bee Colony Losses and What Is Colony Collapse Disorder (CCD)?

**Fig 1:**
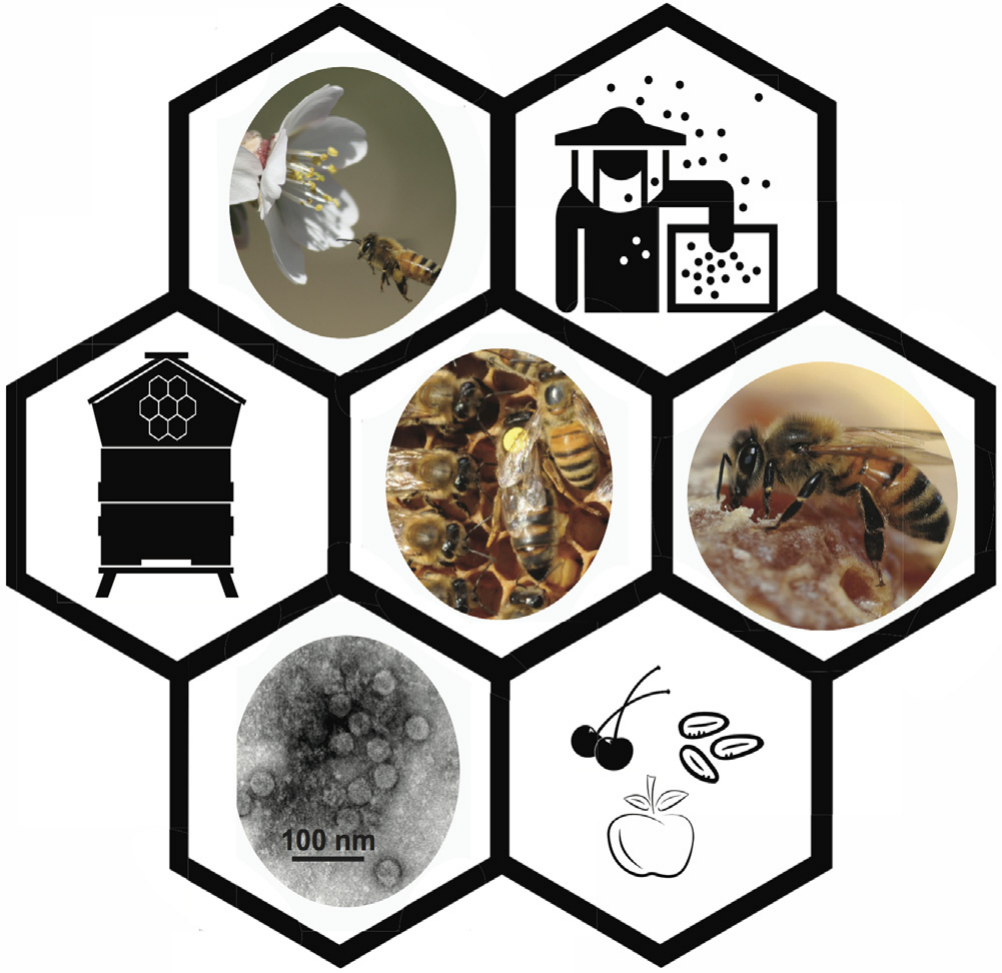
Honey bees are important plant pollinators that are readily infected with RNA viruses. Honey bees (*Apis mellifera*) are eusocial insects that live in large colonies that are composed of sterile female worker bees (~35,000), hundreds of male bees (drones), and a single reproductive female, the queen bee. Beekeepers manage millions of bee colonies for honey production and pollination of numerous fruit, nut, vegetable, and oilseed crops. High losses of bee colonies in North America and some parts of Europe have led researchers to examine the role of multiple stressors, including RNA viruses, on bee health at both the individual and colony levels. Figure photo credits: honey bee and almond flower (middle left) by Christi Heintz, queen bee and attendants (center) and worker bee and honey (middle right) by Kathy K. Garvey, and Lake Sinai virus 2 (LSV2) transmission electron microscope image (lower left) by Sue Brumfield. Noun Project icon credits: bee hive (middle left) by Les vieux garcons, beekeeper (top right) by Luis Prado, and cherry, almonds, and apple by May Irine, Thomas Knopp, and Andrea Bobadilla, respectively.

High annual losses of honey bees, as well as range reductions and local extinctions of wild and native pollinator species, are concerning because bees are important plant pollinators [1]. Approximately one-third of the typical Western diet requires bee pollination, and honey bees (*Apis mellifera*) are the primary pollinators of numerous food crops, including fruits, nuts, vegetables, and oilseeds [2]. Annually, insect-pollinated crops are valued at approximately US$175 billion worldwide and US$17–US$18 billion in both North America and the European Union [3]. The largest agricultural pollination event in the world occurs each February in the Central Valley of California, where nearly 80% of the world’s almonds are produced [4]. This single pollination event requires that over 60% of the commercially managed honey bee colonies in the United States (~1.6 million) be transported to the California almond groves each year (Fig 1) [5]. Since 2006, US honey bee colony losses have averaged 33% annually (increased from ~12% historic level) [6,7]. A small percentage of these losses (3%–8%) are attributed to Colony Collapse Disorder (CCD), which is defined by a specific set of criteria: rapid loss of adult bees, presence of queen and developing bees, delayed pest invasion, and absence of field-diagnosable bee pathogens, including bacterial foulbrood diseases and overwhelming mite infestation [6,8]. Compared to non-CCD affected or healthy colonies, CCD-affected and collapsing colonies have a greater prevalence of pathogens [8], including viruses such as Israeli acute paralysis virus (IAPV) [9], Acute bee paralysis virus (ABPV), Kashmir bee virus (KBV) [10], and Deformed wing virus (DWV) [11,12], though the specific pathogens vary in different sample cohorts (reviewed in [13]). High annual losses have also been reported in some parts of Europe and Asia, though losses vary by year [14]. Despite high annual losses, US beekeepers have maintained a population of ~2.5 million honey bee colonies by dividing their colonies more frequently to make up for colony deaths [5]. Globally, the stock of managed honey bee colonies has increased by 45% from 1961–2007 but has not met the 300% increase in global demand for pollination services [15].

High annual honey bee colony losses in regions that are vital to global food production are unsustainable; therefore, in order to reduce bee colony losses, it is important to identify the factors most responsible. Multiple factors, including pathogens, agrochemical exposure, lack of quality forage, and reduced habitat, negatively impact bee health (reviewed in [1,13,16]). Honey bee colony health is typically evaluated by estimating the population of the colony, which is a superorganism comprising approximately 35,000 sterile female workers (diploid sisters), hundreds of male bees (haploid drones), and a single reproductive queen bee that lays over 1,000 eggs per day during the summer months. Scientists throughout the globe are investigating the relationship between honey bee colony health and multiple abiotic and biotic factors, including viruses.

## What Are the Most Common Viruses Infecting Honey Bees and How Do They Impact Bee Health?

Single-stranded positive sense RNA viruses make up the largest group of honey bee-infecting pathogens. These viruses include the following: the Dicistroviruses, Israeli acute paralysis virus, Kashmir bee virus, Acute bee paralysis virus, and Black queen cell virus; the Iflaviruses, Deformed wing virus, Kakugo virus, Varroa destructor virus-1, Sacbrood virus, and Slow bee paralysis virus; and taxonomically unclassified viruses, Chronic bee paralysis virus, and the recently discovered Lake Sinai viruses, which are a phylogenetically unique, globally distributed group of viruses, including LSV1-7 and other variants (reviewed in [17,18]). RNA viruses are readily detected in bee samples obtained from colonies of varying health and viral presence, and abundance varies by season and geographic location [7,19–21] (reviewed in [13]). Colony level studies have associated virus (i.e., IAPV, KBV, ABPV, DWV, and LSV2) abundance with CCD-affected or weak colonies, though these associations are not universally observed (reviewed in [13]). At the individual bee level, virus infections may result in physical deformities, paralysis, or death (reviewed in [13]). To date, only one double-stranded DNA virus with generally weak pathogenicity (i.e., Apis mellifera filamentous virus) has been described [22].

## How Are Bee Viruses Transmitted?

Honey bee viruses are transmitted both horizontally and vertically (i.e., from queen bees to offspring) [23]. Virus transmission within a colony is enhanced by crowded conditions and the routine transfer of nectar, pollen, and bee bread between colony members via mouth-to-mouth feeding (trophallaxis) [23]. Several viruses have been detected in pollen and honey, and virus infections have been transferred from these substrates to uninfected queen bees and subsequently detected in eggs and progeny [24].

The ectoparasitic mite *Varroa destructor* is a major vector of honey bee viruses, including DWV, VDV-1, and IAPV. *Varroa destructor* mites were first identified in Asia as a parasite of *A*. *cerana* (Eastern honey bee) and later introduced into Europe (1960–70s) and North America (1980s) (reviewed in [25]). *Varroa destructor* mite infestation is a primary cause of colony loss, as mites feed on developing larvae and adults and may kill enough individual bees to cause colony death [25]. The synergistic negative effects of mite infestation and virus infection are best studied for DWV strains and closely related VDV-1 (84% nucleotide identity). The impact of mite infestation on DWV strain diversity was examined in the Hawaiian islands, where comparative analyses of honey bee samples from colonies located on islands that had experienced two or more years of mite exposure had reduced DWV strain diversity, as compared to samples obtained from colonies located on islands that lacked *Varroa destructor* mites [26]. Likewise, experiments performed in the United Kingdom demonstrated that, as compared to oral inoculation, mite-mediated transmission reduced DWV virus strain heterogeneity [27]. In addition, increased replication of a DWV/VDV-1 recombinant strain associated with increased rates of wing deformity and shortened abdomens [27]. The interactions between mites, viruses, and the honey bee immune system remain largely uncharacterized. Some studies suggest that mite infestation modulates host immune function [28,29], whereas other studies indicate that immune gene expression is not perturbed at the transcriptional level [27,30]. Although the mechanisms are not fully understood, mite infestation coupled with viral infection is common and often associated with colony death [10,31]; therefore, beekeepers employ various management strategies to reduce mite levels and mite-associated viral infections.

While bee-infecting viruses are primarily studied in honey bees, and thus upon discovery are called “honey bee viruses,” many of these viruses infect other bee species [24,32,33]. Intra-species and inter-species transmission of bee viruses occurs in natural settings where viruses are spread via floral resources [24,34]. The first study that examined this phenomena detected bee viruses in 11 non-*Apis* hymenopteran species obtained near honey bee colonies and implicated inter-species transmission by phylogenetic analyses of viral sequences, which did not cluster by host species [24]. Furthermore, greenhouse studies demonstrated IAPV transmission between honey bees and bumble bees (*Bombus impatiens*) via shared food sources [24]. Similarly, larger-scale studies have shown that sympatric honey bee and bumble bee populations in Great Britain and the Isle of Man harbored similar virus strains [34,35]. Bumble bees (both managed and native species) and other native or wild pollinators are also very important for pollination of plants in both agricultural and non-agricultural landscapes [36]. Therefore, further investigations of virus host range and the dynamics of intra- and inter-species transmission are required to better understand the role of viruses on bee health, colony losses, range reductions, and local extinctions.

## What Are the Mechanisms of Honey Bee Antiviral Defense?

Bees exhibit pathogen defense mechanisms at both the colony and individual levels. Colony level or social immunity includes behaviors such as grooming and removal of infected and/or mite-parasitized larvae [37]. Individual bee immune mechanisms include phagocytosis, melanization, nodulation, autophagy, as well as signal transduction cascades, including the Jak/STAT, Imd, and Toll pathways, which are activated downstream of pathogen recognition receptor binding of pathogen-associated molecular patterns (PAMPs) (reviewed in [17]). These immune pathways have been implicated in honey bee antiviral defense, but understanding the mechanism(s) of these responses is an active area of research.

RNA interference (RNAi) plays an important role in insect antiviral defense [38]. RNAi is a sequence-specific, post-transcriptional gene and virus silencing mechanism that is induced by double-stranded RNAs (dsRNAs), which are cleaved by a Dicer endonuclease into small inhibitory RNAs (siRNAs) [39]. These siRNAs serve as sequence-specific guides for the RNA-induced silencing complex (RISC) to locate and destroy cognate RNA molecules, including viral RNAs [39]. The role of RNAi in insect antiviral defense has been best characterized in solitary insects including *Drosophila melanogaster*, because of the availability of mutant fly lines, and *Aedes aegypti* and *Anopheles gambi* mosquitos [39]. Numerous studies indicate that RNAi functions in both honey bee gene regulation [40,41] and antiviral defense (reviewed in [17]). The most definitive studies demonstrated that virus abundance was reduced in infected bees or larvae that were treated with virus-specific dsRNAs or siRNAs [42] (reviewed in [17]). Furthermore, small RNAs isolated from naturally infected honey bees were characterized via Northern blot and sequencing [43]. Colony level studies were also performed, though they focused on the effect of virus-specific dsRNAs on colony metrics (i.e., population and honey production) [44]. The use of virus-specific dsRNAs or siRNAs to treat virus infections is currently being investigated; thus, it is important to better understand their impact on bee physiology [45]. Unlike the *Drosophila* model [46], treatment of virus infected bees with non-sequence—specific dsRNAs reduced virus abundance [47,48], suggesting that bees may also have a generalized antiviral immune response that is triggered by dsRNA, akin to the interferon response in mammals (reviewed in [17,45]).

In order to better characterize honey bee antiviral defense mechanisms, several studies examined transcriptional responses to virus infection [47,49–51]. These studies suggest immune pathways including RNAi, Toll, Jak/Stat, Imd, and autophagy play a role in honey bee antiviral defense, though antiviral transcriptional responses varied, likely due to differences in experimental factors. Therefore, additional studies that control for age, inoculum route, and virus are required to discern universal, age-dependent antiviral responses and virus-specific antiviral responses. Similar to other co-evolving host—pathogen relationships, honey bee viruses have likely evolved mechanisms to overcome and/or evade bee immune responses. For example, several honey bee infecting Dicistroviruses (i.e., IAPV, KBV, and ABPV) encode a DvExNPGP motif at the 5′ terminus of their genomes, suggesting these honey bee-infecting viruses encode putative viral suppressors of RNAi similar to the Dicistroviruses Cricket paralysis virus and *Drosophila* C virus [52]. In addition, viruses may manipulate host gene expression to enhance virus entry, replication, and/or exit. A more complete understanding of honey bee antiviral defense and viral counter-measures may lead to the development of strategies that reduce virus infection in bee colonies.

## What Is the Future of Honey Bee Virology?

Honey bee virology is a rapidly growing field currently in its infancy. Researchers are employing advanced colony level tools (e.g., imaging and computational image analysis) and molecular level techniques, including high-throughput sequencing, bisulfite sequencing, and qPCR, to investigate the impact of viruses on bee health at the colony, individual, and cellular levels. Cultured cells, both primary cells and immortalized cell lines, provide the opportunity to study host—pathogen and pathogen—pathogen dynamics while minimizing confounding factors (e.g., additional pre-existing conditions, outbred bee populations) inherent to performing in vivo studies that utilize honey bees obtained from managed colonies in natural settings. Recently, an immortalized honey bee cell line (AmE-711) was utilized for the first time to investigate host—virus and virus—virus interactions in mixed infections [53]. Both in vitro and in vivo experiments determined that IAPV outcompeted other viruses (i.e., SBV, DWV, and BQCV) in mixed infections; although, when KBV was in the initial inoculum, it outcompeted IAPV [53]. To date, there are no infectious honey bee virus clones. Though studies that utilize virus preparations from infected pupae and model viruses are informative, both are associated with the advantages and disadvantages of these systems. Therefore, current efforts to produce infectious clones of honey bee viruses that can be quantified and manipulated in vitro will advance the field.

In conclusion, this is an exciting time in honey bee virology. Important research topics include (1) understanding honey bee antiviral responses, including those triggered by dsRNA and siRNA, at the cellular and molecular levels, and identifying viral counter measures; (2) identifying the most pathogenic virus strains and determining what factors govern their virulence and transmission; (3) investigating the role of the bee microbiome on virus infection and bee health; and (4) determining how synergistic variables, including agrochemical exposure and nutritional stress, impact viral pathogenesis. Further investigation of these and other topics will advance our understanding of bee biology, host—pathogen interactions, colony health, and may lead to the development of strategies that limit colony losses.
